# Father figures

**DOI:** 10.1186/2041-2223-2-21

**Published:** 2011-10-06

**Authors:** Mark A Jobling

**Affiliations:** 1 Department of Genetics, University of Leicester, University Road, Leicester LE1 7RH, UK

## 

Show a portrait of Thomas Jefferson (1743-1826), third US President, to a room full of Brits, and no-one ever knows who he is. But if there is an American there, it's a different matter. They always recognise him: maybe not from his appearance on the two-dollar bill (a Baltimore resident was briefly incarcerated for proffering a wad of these rarities in a car radio shop), but more likely from the ubiquitous nickel, or from high school history lessons.

I know this because of the talks I give to the general public. Speaking to non-scientists about science is fun (as well as part of our solemn duty to obey our munificent funders) and the Jefferson story is a good one to tell, with its ingredients of sex, race, slavery and genetics. Thomas's wife Martha predeceased him by 43 years, and Thomas was true to his word never to marry again. But it was alleged that he had a relationship with one of his slaves, Sally Hemings, and may have been the father of her six children [1].

Genetics entered the picture in 1998 when Eugene Foster, a retired Virginia pathologist, realised that Y-chromosome testing might be able to shed light on the controversy. He traced living male-line descendants of Sally's first and last children, Tom and Eston, and to provide a comparative sample carrying the 'Jefferson' Y chromosome recruited male-line descendants of Thomas's paternal uncle Field Jefferson, since Thomas had no acknowledged sons of his own. The notion was to compare Y chromosomes, and ask if there was a match between Sally's and Thomas's descendants.

We and our colleagues analysed Y-chromosomal markers (SNPs, STRs and a minisatellite) in these samples [2], and showed that Eston's descendant, John Weekes Jefferson, did indeed carry the same Y-type as the Field Jefferson descendants. This finding supported the belief that Thomas fathered Sally's last child. The evidence is strengthened by the great rarity of the Y chromosome, now known to belong to haplogroup T1a* [3] - the sharing is unlikely to be a coincidence. Of course, a weakness of any such Y-chromosome analysis is that any other man in Thomas's patriline (his brother Randolph, for instance) would have carried the same Y, and the two possible fathers cannot be formally distinguished by DNA analysis. However, historians helped here, by providing the telling circumstantial evidence that Thomas and Sally were together at Monticello nine months before the birth of each of her children.

But what of the descendants of Tom, Sally's first son? Here, there were more twists to the tale: the family had a cherished and widely believed oral history of their descent from the President, and this was dashed when the results were published [4], showing that they do not carry the Jefferson lineage. Another unwelcome revelation was the fact that one of the tested descendants carried a different Y-chromosome type from his putative first cousin - so a recent non-paternity had occurred. These painful findings illustrate two of the potential pitfalls of genealogical analysis, made worse by the fact that they were communicated to the family not through a confidential discussion, but in a phone call from *US News and World Report*, thanks to the hasty publication of the paper. The indecent rush was due to the topical and (to some) seemingly parallel presidential saga of Bill Clinton and Monica Lewinsky. Bill's middle name, incidentally, is Jefferson. Whoever said that scientific journals were only interested in science?

Although it revealed a non-paternity event, the success of the Jefferson study relied upon non-paternity being rare - if it had been much more common than the 1 event in 67 transmissions observed (~1.5% per generation), then no reliable conclusions could have been reached. Yet there is a wide perception that the frequency is indeed much higher than this, and an 'urban mythical' figure of 10% is commonplace [5]. Genetic studies in western Europe contradict this high value: Swiss pedigrees show < 1% [6], and data from UK cystic fibrosis screening show 1.35% [7]. Hollingsworth's examination of the genealogies of the British peerage between 1550 and 1949 [8] counted 23,724 legitimate sons and 196 male 'bastards' - ~0.8% overall. There is much variation around this average, with the prolific first Earl Ferrers (1650-1717) siring 30 bastards among his 57 offspring. But frequencies also vary greatly from place to place, depending on customs and socioeconomic circumstances. For example, among the Himba, a semi-nomadic people in northwest Namibia, 17.6% of all children born within marriage were not fathered by the husband [9].

DNA analysis, typically using the same autosomal STRs as are used in individual identification, generally provides a straightforward answer to any question about paternity when the child, mother and putative father are analysed. The paternity testing industry is large - ~415,000 tests were done in the USA in 2008 - and provides much interesting data for the curious geneticist, including fabulously detailed information about allele- and sex-specific mutation rates of STRs. But what paternity testing data cannot provide is a useful estimate of the non-paternity frequency; when a test is done, there's often a prior reason to suspect some irregularity, and published frequencies from these sources range from 14% to over 50% [10].

DNA-based kinship analysis is also used in the controversial practice of familial searching, where a crime-scene DNA profile is used to search a database for profiles sufficiently similar to derive from close relatives; siblings, for example, are expected to share on average half of their DNA by descent. Familial searching has been most used in the UK, and has had some famous successes. Craig Harman was convicted of manslaughter after his profile on a brick led police to his brother, and thence to him. Serial rapist David Lloyd's DNA profile picked out that of his sister, who entered the National DNA Database following a driving offence. Ethical objections have been raised, because familial searching increases police interest in individuals based purely on their relatives' previous contact with the criminal justice system, but nonetheless the method seems to be catching on [11].

We proposed another controversial way of discovering suspects from crime-scene DNA, by predicting their surnames from a large database of Y-STR profiles and associated names [12]. Forensic databases of this kind do not yet exist, but genetic genealogy companies maintain publicly accessible databases with many tens of thousands of entries (e.g. http://www.ysearch.org). How well it would work would depend on the simplicity of the link between ancestral founders of heritable surnames and their modern descendants. For some rare surnames, among them Attenborough, it is simple - over 80% of apparently unrelated Attenborough men share a Y haplogroup, and a set of closely related Y-STR haplotypes [13]. If we assume that the common Y type descends from the original Mr Attenborough when the name was founded about 700 years ago, and that the other Y lineages are due to non-paternity events, then this allows us to calculate an average non-paternity frequency of 1-4% per generation.

Jeffersons, however, are not like this. Among 85 unrelated men with this surname there are many different Y chromosome types, reflecting as many as 10 different possible founders. But much to their surprise, two unrelated English Jeffersons share the rare hg T1a* haplotype with the President [14]. Luckily, these distant relatives link Thomas Jefferson to Britain within the last few centuries, rather than to the heartland of haplogroup T, the Middle East, thus defusing yet another possible mystery story about the celebrated figure on the $2 bill.
